# Macrophage-specific RhoA knockout delays Wallerian degeneration after peripheral nerve injury in mice

**DOI:** 10.1186/s12974-021-02292-y

**Published:** 2021-10-15

**Authors:** Jiawei Xu, Jinkun Wen, Lanya Fu, Liqiang Liao, Ying Zou, Jiaqi Zhang, Junyao Deng, Haowen Zhang, Jingmin Liu, Xianghai Wang, Daming Zuo, Jiasong Guo

**Affiliations:** 1 Department of Histology and Embryology, School of Basic Medical Sciences, Southern Medical University, Guangzhou Ave North 1838, Guangzhou, 510515 China; 2 Guangdong Provincial Key Laboratory of Construction and Detection in Tissue Engineering, Southern Medical University, Guangzhou, 510515 China; 3 Department of Neurology, Jiangmen Central Hospital, Affiliated Jiangmen Hospital of Sun Yat-Sen University, Jiangmen, 529030 China; 4 Bioland Laboratory (Guangzhou Regenerative Medicine and Health Guangdong Laboratory), Guangzhou, 510700 China; 5 Department of Medical Laboratory, School of Laboratory Medicine and Biotechnology, Southern Medical University, Guangzhou, 510515 Guangdong China; 6 Department of Spine Orthopedics, Zhujiang Hospital, Southern Medical University, Guangzhou, 510280 China; 7 Key Laboratory of Mental Health of the Ministry of Education, Guangdong-Hong Kong-Macao Greater Bay Area Center for Brain Science and Brain-Inspired Intelligence, Guangdong Province Key Laboratory of Psychiatric Disorders, Guangzhou, 510515 China

**Keywords:** RhoA, Macrophage, Wallerian degeneration, Nerve regeneration, Peripheral nerve injury

## Abstract

**Background:**

Plenty of macrophages are recruited to the injured nerve to play key roles in the immunoreaction and engulf the debris of degenerated axons and myelin during Wallerian degeneration, thus creating a conducive microenvironment for nerve regeneration. Recently, drugs targeting the RhoA pathway have been widely used to promote peripheral axonal regeneration. However, the role of RhoA in macrophage during Wallerian degeneration and nerve regeneration after peripheral nerve injury is still unknown. Herein, we come up with the hypothesis that RhoA might influence Wallerian degeneration and nerve regeneration by affecting the migration and phagocytosis of macrophages after peripheral nerve injury.

**Methods:**

Immunohistochemistry, Western blotting, H&E staining, and electrophysiology were performed to access the Wallerian degeneration and axonal regeneration after sciatic nerve transection and crush injury in the *Lyz*^*Cre*+*/−*^; *RhoA*^*flox/flox*^ (cKO) mice or *Lyz2*^*Cre*+*/−*^ (Cre) mice, regardless of sex. Macrophages’ migration and phagocytosis were detected in the injured nerves and the cultured macrophages. Moreover, the expression and potential roles of ROCK and MLCK were also evaluated in the cultured macrophages.

**Results:**

1. RhoA was specifically knocked out in macrophages of the cKO mice; 2. The segmentation of axons and myelin, the axonal regeneration, and nerve conduction in the injured nerve were significantly impeded while the myoatrophy was more severe in the cKO mice compared with those in Cre mice; 3. RhoA knockout attenuated the migration and phagocytosis of macrophages in vivo and in vitro; 4. ROCK and MLCK were downregulated in the cKO macrophages while inhibition of ROCK and MLCK could weaken the migration and phagocytosis of macrophages.

**Conclusions:**

Our findings suggest that RhoA depletion in macrophages exerts a detrimental effect on Wallerian degeneration and nerve regeneration, which is most likely due to the impaired migration and phagocytosis of macrophages resulted from disrupted RhoA/ROCK/MLCK pathway. Since previous research has proved RhoA inhibition in neurons was favoring for axonal regeneration, the present study reminds us of that the cellular specificity of RhoA-targeted drugs is needed to be considered in the future application for treating peripheral nerve injury.

## Background

RhoA is a pivotal regulatory molecule for cytoskeletal remodeling in various cells [1–3] and has been considered as an important target in regulating axonal regeneration [4, 5]. In the axotomized neuron, RhoA is activated and then plays roles in inducing both the collapse of axonal growth cone and the death of the lesion neurons through its downstream effector ROCK. Therefore, inhibitors of RhoA or ROCK are commonly utilized to promote peripheral nerve regeneration [6, 7]. However, considering that the drugs used to treat the injured nerve may not just affect neurons but also impact on other cells, and a certain molecule might play diverse roles in different cell types [8–10]. Hence, we believe that revealing the role of RhoA in different cells is necessary. Recently, our team has found several surprising effects of RhoA on Schwann cells, such as RhoA knockdown depressing Schwann cell’s proliferation, migration, and myelination, and the typical downstream molecule ROCK does not contribute to some effects of RhoA on Schwann cells. For example, RhoA regulates Schwann cell’s differentiation through JNK pathway, while influencing its proliferation by regulating AKT pathway [8, 10].

After peripheral nerve injury (PNI), abundant blood-derived macrophages are recruited to the injured area [11, 12]. These infiltrated macrophages engulf and remove the debris of axon and myelin to accelerate Wallerian degeneration and thus provide a favorable microenvironment for the following nerve regeneration [13, 14]. Once RhoA-targeted drugs were administrated to treat the injured nerve, the drugs must have by-effects on macrophages through regulating their cytoskeleton. However, the influence of RhoA inhibition in macrophages on the process of Wallerian degeneration and nerve regeneration after PNI remains elusive. Since previous reports have presented conflicting opinions on the roles of RhoA in the biofunctions of macrophages [15, 16], and RhoA inhibition in macrophages in different tissues might result in different outcomes [17–20], we believe that macrophage-specific RhoA knockout mice are needed for elucidating the bioeffects of RhoA on macrophage in the injured nerve, and we hypothesize that RhoA might influence Wallerian degeneration and nerve regeneration by affecting the migration and phagocytosis of macrophages after PNI.

Herein, using a line of *Lyz*^*Cre*+*/−*^; *RhoA*^*flox/flox*^ mice, we demonstrated that macrophage-specific RhoA knockout significantly retarded Wallerian degeneration and nerve regeneration after PNI, which probably due to decreased migration and phagocytosis of macrophages after RhoA knockout. Furthermore, RhoA regulates the migration and phagocytosis of macrophages via the ROCK/MLCK pathway. As previous studies have proved inhibiting RhoA in neurons was favor for axonal regeneration, the slower Wallerian degeneration and poor nerve regeneration in macrophage-specific RhoA knockout mice revealed by present study reminds us that the cellular specificity of RhoA-targeted drugs is needed to be considered in the future applications for PNI.

## Methods

### Animals

All mouse lines were maintained on a C57BL/6J background and housed under standard conditions (22 ± 1 °C) in a specific pathogen-free animal facility with a 12/12-h light–dark cycle with water and food ad libitum at Southern Medical University. The Institutional Animal Care and Use Committee of Southern Medical University, P.R. China approved all animal experiments. All efforts were made to minimize the number of animals and their suffering. B6.129P2-Lyz2tm1(cre)Ifo/J mice, obtained from Shanghai Model Organisms Center, Inc. (Shanghai, China), has a nuclear-localized Cre recombinase inserted into the first coding ATG of Lyz2 for abolishing endogenous Lyz2 gene function and NLS-Cre expression under control of the endogenous Lyz2 promoter/enhancer element [21]. *RhoA*^*flox/flox*^ mice obtained from Cyagen company (Suzhou, China) were crossbred with B6.129P2-*Lyz2*^*tm1(cre)Ifo*^/J mice to generate *Lyz*^*Cre*+*/−*^*; RhoA*^*flox/flox*^ mice with monocyte/macrophage lineage-specific RhoA conditional knockout, and genotyped as described in Liu et al. [22].

### Animal surgery

Adult mice (8 weeks, regardless of sex) were anesthetized with an intraperitoneal injection of 12 mg/mL tribromoethanol (180 mg/kg body weight), and then the right sciatic nerve was bluntly exposed. A crush injury at 0.3 cm distal to the sciatic notch was performed using a fine, smooth and straight hemostat (#EMZ-125-Z, Ermei, China) for 2 min, which established a sciatic nerve crush injury model [23]. A sciatic nerve transection injury model was established through a transection at 0.3 cm distal to the sciatic notch. For the sham operation, the left sciatic nerve was only exposed without crush or transection. All animals received analgesic treatment with buprenorphine (0.05 mg/kg) [24, 25] injection twice a day until 3 days post the surgery. Based on the protocols of power calculation for group size [26, 27] and the data of pre-experiment, 6 mice/group were used for each test.

### Culture of bone marrow-derived macrophages and pharmacological treatment

Macrophages were isolated and cultured as previously described [28, 29]. Briefly, bone marrow cells were flushed out with Dulbecco’s modified Eagle’s medium (DMEM)/F12 (Gibco, 11330057) from the tibia and femur of the control (*Lyz2*^*Cre*+*/−*^, described as Cre below) and conditional RhoA-deleted (*Lyz2*^*Cre*+*/−*^*; RhoA*^*flox/flox*^, described as cKO below) mice, regardless of sex, after euthanasia. Then the bone marrow cells were cultured for 7 days in DMEM/F12 containing 10% fetal bovine serum (FBS, Sigma, F8318), antibiotics (100 U/mL penicillin, 100 mg/mL streptomycin, Gibco, 15140122), and 10 ng/mL macrophage colony stimulating factor (MCSF, R&D, 416-ML-010) at 37 °C and under 5% CO_2_.

To investigate the functions of ROCK and MLCK in the macrophage's migration and phagocytosis, 10 μM Y27632 (Selleck, S1049) [16] and 25 μM ML7 (Abcam, AB120848) were added into the cultured medium in wide type macrophages and maintained for 24 h.

### Myelin debris preparation

Myelin debris was produced using mouse brains and spinal cords as described elsewhere [31]. In brief, brains and spinal cords were isolated from wild-type mice aged 8–10 weeks after euthanasia by cervical dislocation and then shattered into tiny particles by sonication. Tissue debris was rinsed with Milli-Q water three times by centrifugation for 15 min at 4 ℃ at 14,462 g. Finally, myelin debris was resuspended with Hank’s balanced salt solution (HBSS, Gibco, C14175500BT) at a concentration of 100 mg/mL and stored at − 80℃.

### Phagocytic capability assay

Phagocytosis in macrophages was assessed by the ingestion of lumispheres [32] and myelin debris. For better staining and observation, macrophages were seeded on the cell slides in 24-well plates at a density of 1 × 10^5^ cells/well. The next day, 0.1 mg/mL fluorescent lumispheres (1 µm diameter, BaseLine Chromtech, 7-3-0100) or 1 mg/mL myelin debris were added into the culture for 3 h or 24 h, respectively. After being rinsed three times with HBSS to remove the attached lumispheres or myelin debris from the cell surface, the cells were fixed with 4% paraformaldehyde (PFA, Macklin, P804536) and stained with F4/80 (1:300, Abcam, ab6640) to identify the outlines of the macrophages. The number of lumispheres ingested by each cell was counted under a fluorescent microscope (Leica). To observe the ingestion of myelin debris, F4/80 was co-stained with Oil Red O (ORO, Solarbio, G1262) and MBP (1:100, Biolegend, SMI-99), respectively.

### Migration assay

The migration of the macrophages was assessed by a Transwell assay using 6.5-mm Transwell chambers (8-µm pores, Corning Costar, 3422) as described previously [32, 33]. After the chambers were pretreated with 0.1 mg/mL poly-l-lysine hydrobromide (PLL, Sigma-Aldrich, P1274) solution or culture medium, 1 × 10^5^ macrophages in 100 µL of DMEM/F12 containing 1% FBS were seeded into the upper chamber, and the lower chamber was filled with 600 µL DMEM/F12 containing 10% FBS and 1 mg/mL myelin debris without cells. The macrophages were allowed to migrate for 18 h, and then fixed with 4% PFA for 20 min. After careful removal of the cells on the upper surface with a cotton swab, the cells adhered to the lower surface of the Transwell membrane were stained with 0.1% crystal violet (Leagene, DZ0055) for 30 min. Then, five images of each membrane (the center and four quadrants) were captured under an inverted microscope (Leica) for quantification.

### Frozen section and immunohistochemistry

Sciatic nerves and gastrocnemius were harvested from mice after transcardial perfusion with 4% PFA and post-fixed in 4% PFA for 24 h. After dehydration in 30% sucrose overnight, the nerves were embedded in Optimum Cutting Temperature Compound (OCT, Sakura, 4583) for frozen sectioning and the following immunofluorescence staining. Immunohistochemistry was performed as follows. Followed by permeabilization with 0.5% Triton X-100 (Sigma, X100) for 30 min, the samples were blocked with 5% fish gelatin (Sigma, G7041) containing 0.3% Triton X-100 at room temperature (RT) for 1 h, then incubated with the primary antibodies diluted in blocking buffer at 4 ℃ overnight. Alexa 488 and/or 568 fluorescent-conjugated secondary antibodies were applied at RT for 2 h. Samples were incubated with 1 µg/mL 4′,6-diamidino-2-phenylindole (DAPI, Sigma, D8417) for 2 min to counterstain cell nuclei [34]. The primary antibodies used for immunofluorescence staining were as follows: mouse anti-MBP (1:100, Biolegend, SMI-99), rabbit anti-NF200 (1:400, Sigma, N4142), rabbit anti-P0 (1:100; Millipore, ABN363), rabbit anti-GAP43 (1:400, Abcam, ab16053), rat anti-F4/80 (1:400, Abcam, ab6640), mouse anti-RhoA (1:200; Santa, sc-418). The secondary antibodies used for immunofluorescence staining were as follows: Alexa 568-conjugated goat anti-rabbit (1:400, Invitrogen, A-11011), Alexa 488-conjugated goat anti-rabbit (1:400, Invitrogen, A-11008), Alexa 488-conjugated goat anti-mouse (1:400, Invitrogen, A-11001), Alexa 568-conjugated goat anti-mouse (1:400, Invitrogen, A-11004), Alexa 488-conjugated goat anti-rat (1:400, Invitrogen, A-11006), Alexa 568-conjugated goat anti-rat (1:400, Invitrogen, A-11077).

To quantify the regenerating axons in the distal trunk of the injured nerve, 3 vertical lines were placed at 2, 4, and 6 mm distal to the injury site, the number of GAP43 positive axons crossing these lines was measured [35]. To evaluate the inflammation around the injury site, a rectangle image with a size of 1 mm × 2 mm was captured in the central of distal trunk close to the injury site, and all the F4/80 positive macrophages in the image were counted.

### Oil Red O (ORO) staining

For assessing the degradation of myelin during Wallerian degeneration and the phagocytosis of macrophage, ORO staining was performed [36]. Briefly, the ORO staining solution was prepared as a mixture of 0.3% ORO (dissolved in 60% 2-propanol, Solabio, G1262) and deionized water (ratio 3:2). After being rinsed with 0.01 M PBS and 60% isopropanol, the sections were incubated in the ORO solution at 37 ℃ for 15 min and then rinsed in 60% isopropanol and 0.01 M PBS before routine mounting.

### Western blotting

For Western blotting, subjected cells were washed twice with ice‐cold PBS, scraped, and lysed in a RIPA buffer (Fdbio science, FD009) containing a protease inhibitor cocktail (1:100, Fdbio, FD1001). The sciatic nerve tissues were (with length 7 mm) dissected from the distal trunk of injured nerves were quickly frozen in liquid nitrogen for 30 s before being pulverized and lysed in a RIPA buffer containing a protease inhibitor cocktail. Lysates were incubated on ice for 30 min and centrifuged (14,462×*g*, 20 min, 4 °C) in order to collect the supernatant. Extracts were separated in an SDS‐PAGE sample buffer (Fdbio science, FD006) on 10% SDS‐PAGE gels and transferred to a polyvinylidene difluoride membrane (PVDF, Millipore, IPVH0010). Blots were blocked with 5% BSA for 1 h and incubated with primary antibodies overnight at 4 °C and then incubated with HRP‐conjugated secondary antibodies at RT for 2 h. The following primary antibodies were used: rabbit anti‐GAPDH (1:2000, Abcam, ab8245), rabbit anti‐mouse anti-RhoA (1:500; Santa, sc-418), GAP43(1:1000, Abcam, ab16053), mouse anti-MBP (1:500, Biolegend, SMI-99), rabbit anti-NF200 (1:1000, Sigma, N4142), rabbit anti-P0 (1:500; Millipore, ABN363), rabbit anti-ROCK2 (1:1000, Abcam, ab125025), rabbit anti-MLCK (1:1000, Abcepta, AP7966A). The secondary antibodies were as follows: goat anti-rat IgG (1:2000, Invitrogen, 31471), goat anti-rabbit IgG (1:2000, Invitrogen, 31460), goat anti-mouse IgG (1:2000, Invitrogen, 32430).

The Western blotting for each protein was repeated three times for each test. Immunoreactive protein bands were detected and imaged by enhanced chemiluminescence (ECL, Millipore, Burlington, MA, USA) using a Lumazone system (Roper, Trenton, NJ, USA). The integrated optical density (IOD) of each lane was quantified with the Image J software, and the expression levels of the targeted proteins were calculated by dividing the IOD of the targeted proteins by the IOD of the GAPDH.

### Electrophysiological test

The electrophysiological test was performed as per the previous studies [35–37]. The animals were anesthetized with an intraperitoneal injection of 12 mg/mL tribromoethanol (90 mg/kg body weight). Then the involved sciatic nerve was exposed, stimulating electrodes were applied to the host nerve trunk 3 mm proximal to the injury site, and a pair of electrodes was placed in the intrinsic foot muscle to record the compound muscle action potential (CMAP) with a set of electrophysiological record (Axon Digidata 1550 Digitizer, Molecular Devices). The amplitude and latency of each animal were recorded at 10 attempts, then the highest amplitude and mean latency were measured and analyzed.

### Evaluation of gastrocnemius myoatrophy

Gastrocnemius muscles harvested from the perfused mice of 4 weeks post-crush injury were evaluated as previously reported [35, 37]. Briefly, the wet weight of each gastrocnemius muscle was measured after the connective tissue was trimmed off and the trimmed muscle was dried by filter paper. The wet weight ratio of the gastrocnemius in the ipsilateral leg of the injured nerve to that of the contralateral leg whose sciatic nerve was not injured was calculated as follows. Wet weight ratio (%) = weight of the injured gastrocnemius/weight of the uninjured gastrocnemius × 100.

A set of sections were prepared from the mid-belly of each gastrocnemius muscle, then transversally sectioned with a thickness of 10 μm for routine hematoxylin–eosin (H&E) staining to show the outline of the myofibers. As described in previous publications [35], six random non-overlapping fields of five sections from each animal were captured, and the area of myofibers in the transverse section and neuromuscular junctions in the longitudinal section were counted by using Image-Pro Plus 6.0 software.

### Statistical analysis

All values are presented as the mean ± standard error of the mean (SEM), and SPSS 23.0 software (IBM, Armonk, NY, USA) was used for the statistical analysis. Differences between two groups were analyzed using Student’s *T*-test. A one‐way ANOVA (Bonferroni post hoc comparison) was used to determine the statistical significance of the differences among multiple groups, and the *p*‐values are indicated by *p < 0.05, **p < 0.01, ***p < 0.001. A *p*-value of < 0.05 was considered statistically significant.

## Results

### Identification of macrophage-specific knockout of RhoA in *Lyz2*^*Cre*+*/−*^*RhoA*^*flox/flox*^ mice

**Fig. 1:**
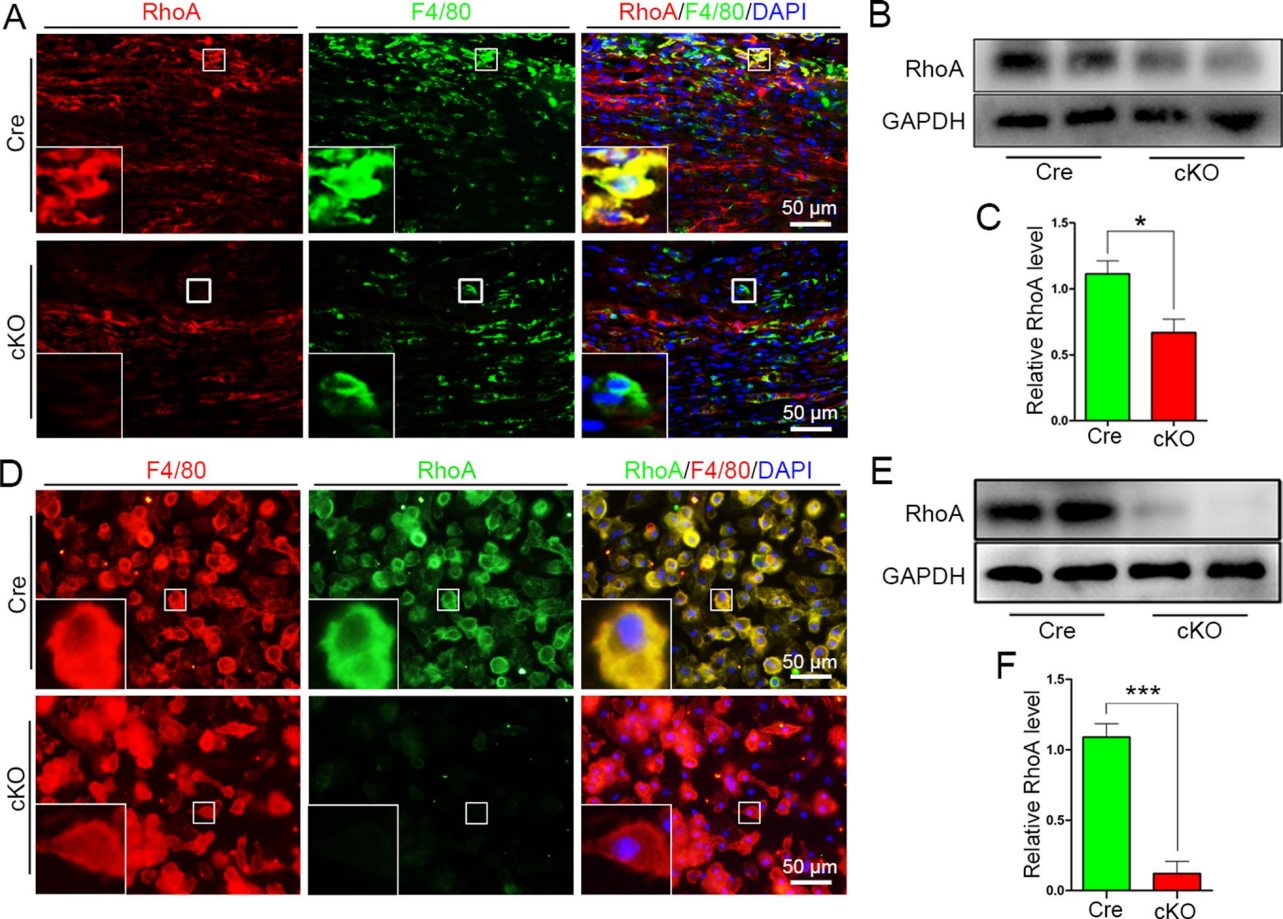
Identification of macrophage-specific knockout of RhoA. **a** F4/80 and RhoA double-immunofluorescent staining in the crush injured sciatic nerve at 7 dpi, which showing that even RhoA is expressed in the injured nerve but is negative in the F4/80^+^ macrophages in the cKO Group. Scale bars: 50 µm. **b**, **c** Western blots and statistical analysis showing the level of RhoA protein in the injured nerve is significantly decreased in the cKO group comparing to the Cre group. **d**–**f** Immunocytochemistry and Western blots showing that RhoA expression is hard to be detected in the isolated macrophages in vitro. Scale bars: 50 µm. The data are presented as the mean ± SEM values (*n* = 6 per group)

To investigate the roles of RhoA in macrophages after PNI, we firstly generated mice with macrophage-specific RhoA knockout under Cre-loxp system. Mice carrying floxed *RhoA* alleles were crossed with mice expressing Cre recombinase under the controlled by *Lyz2* regulatory sequences. The progeny were viable and fertile, with litters born in accordance with the expected Mendelian distributions [19, 22]. We performed double immunohistochemistry with antibodies of F4/80 [38] (a specific marker of macrophage) and RhoA in the injured sciatic nerves at 7 days post-injury (dpi), and results showed that RhoA was negative in F4/80^+^ macrophages in the *Lyz2*^*Cre*+^*RhoA*^*flox/flox*^ (cKO) group while positive in the control *Lyz2*^*Cre*+^*RhoA*^+*/*+^ (Cre) group (Fig. 1a). Western blots showed the level of RhoA protein in the injured nerve is significantly lower in cKO group than that in Cre group (*p* = 0.035, Fig. 1b, c). When the bone marrow-derived macrophages were isolated and cultured in vitro, both immunocytochemistry and Western blotting illustrated that RhoA expression was hardly detectable in the cKO macrophages (Fig. 1d–f). These results indicate that RhoA has been specifically knocked out in macrophages in the cKO mice.

### Macrophage-specific RhoA depletion retards Wallerian degeneration in the injured nerve

**Fig. 2:**
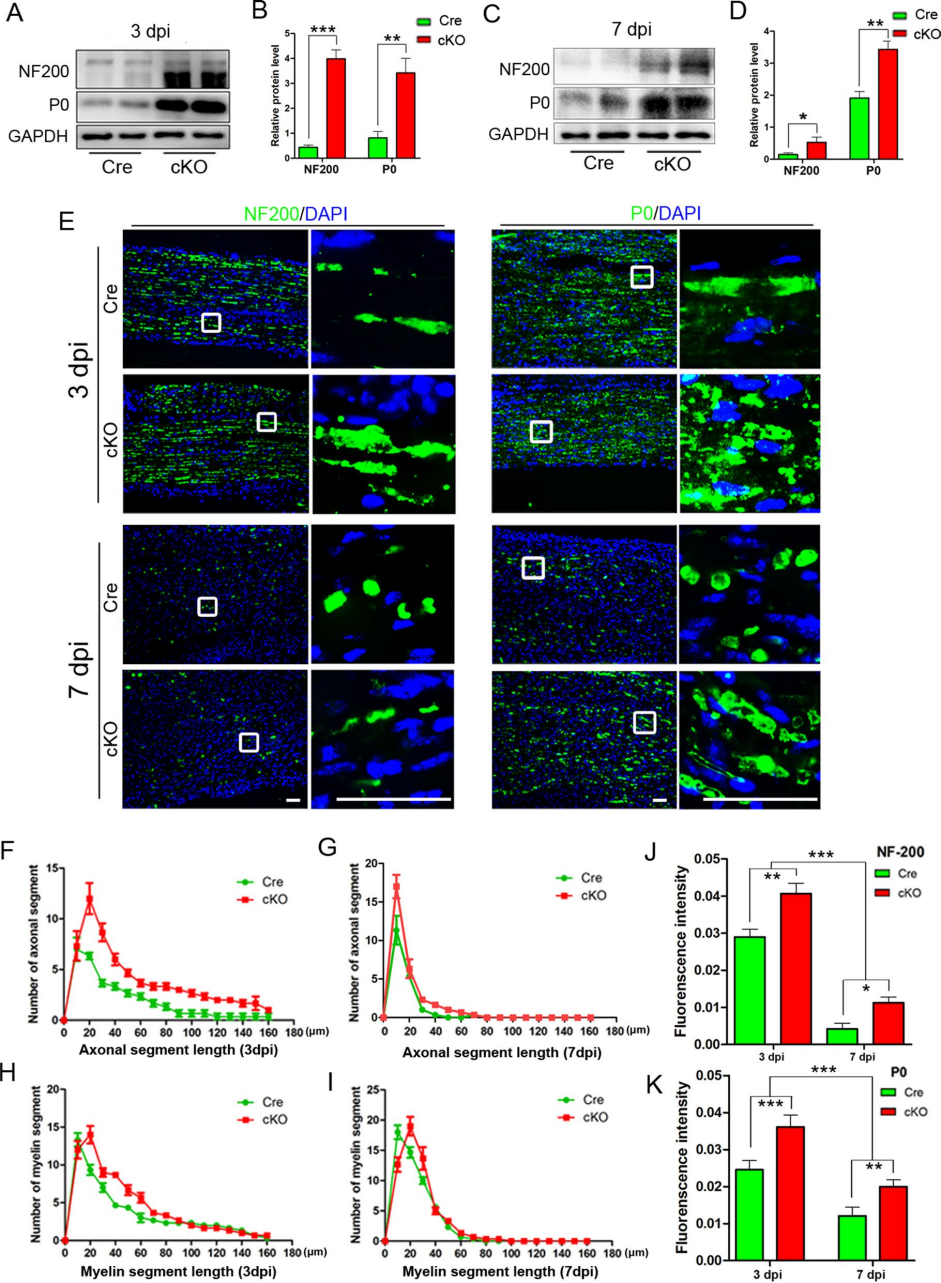
RhoA cKO in macrophages inhibits the degeneration of axons and myelin in the injured nerve. **a**–**d** Western blots and statistical analysis illustrating that the levels of NF200 and P0 protein at 3dpi (**a**, **b**) and 7dpi (**b**, **c**) are significantly higher in cKO group than those of the Cre group. **e** Immunohistochemistry in the longitudinal sections of transected sciatic nerve’s distal trunk, which showing the axons (NF200^+^) and myelin sheaths (P0^+^) are fragmented and get worse from 3 to 7 dpi. **f**–**k** Quantification of the length distributions of axons (**f,** **g**) and myelin fragments (**h**, **i**) as well as the NF200 (**j**) and P0 (**k**) immunointensities showing both the length of axonal fragments and myelin fragments are significantly higher in the cKO group than those of the Cre group at both of 3 dpi and 7 dpi. Scale bars: 50 µm. Data are expressed as the mean ± SEM (*n* = 6 per group)

To evaluate the influence of macrophage-specific RhoA depletion on Wallerian degeneration, sciatic nerves were transected to model the process of Wallerian degeneration in both cKO and Cre mice. At 3 dpi or 7 dpi, the distal segments of the injured nerves were collected to perform immunohistochemistry and Western blotting with antibodies of neurofilament 200 (NF200, a marker of axons) and myelin protein zero (P0, a marker of myelin) to show the degeneration of axons and myelin. The protein expression levels of NF200 and P0 are higher in the cKO group than that in the Cre group, suggesting that more axons and myelin remained in the cKO group (Fig. 2a–d). Immunostaining of longitudinal sections of the injured nerves showed that NF200^+^ axons and P0^+^ myelin were fragmented, and the fragmentation was more severe in the 7 dpi than 3 dpi (Fig. 2e). Based on the quantification as described previously [36, 39], we discovered that the lengths of the axonal and myelin segments in the cKO group were longer than those in the Cre group (Fig. 2f–k).

### Macrophage-specific RhoA depletion attenuates nerve regeneration after nerve injury

**Fig. 3:**
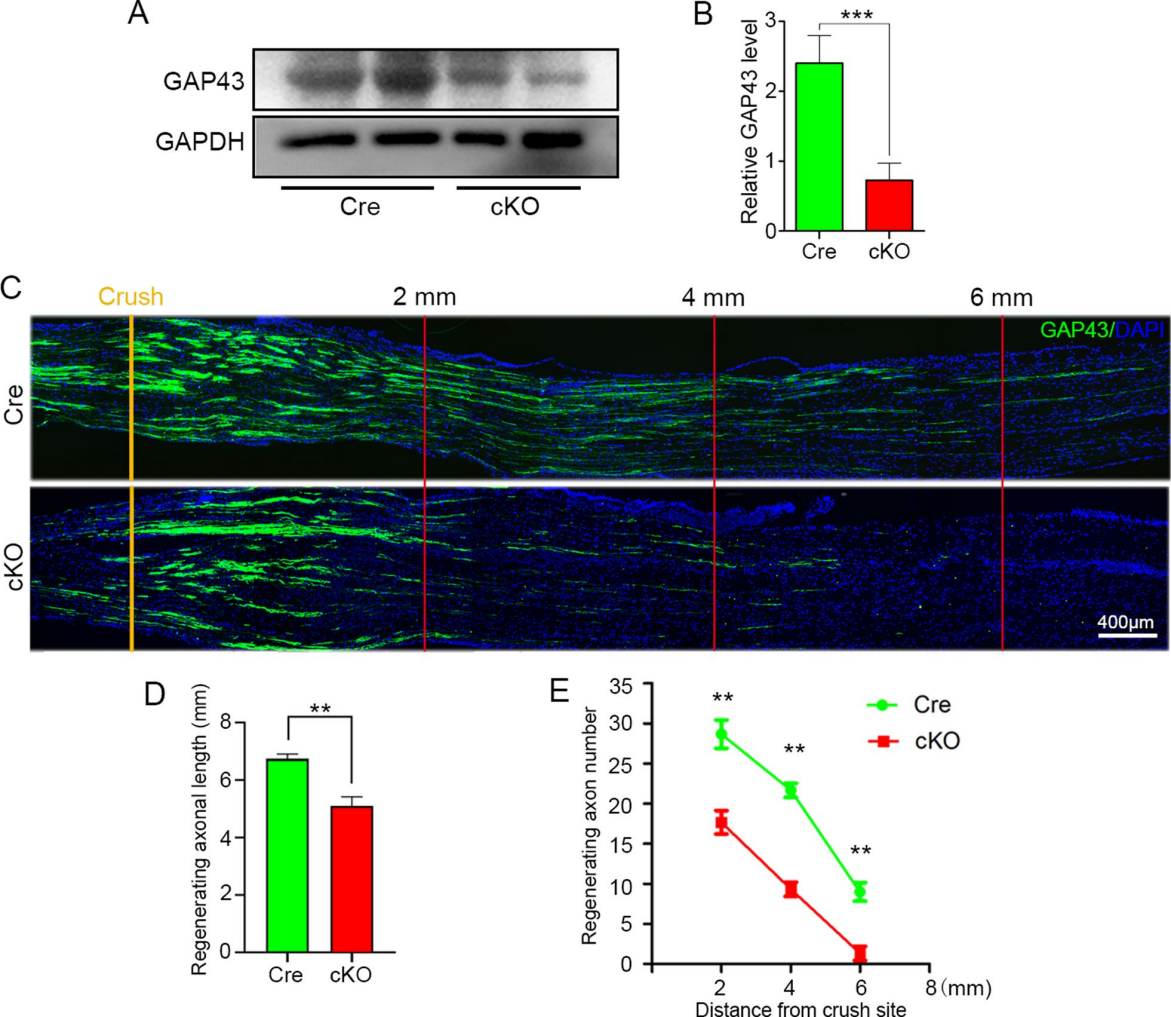
Less and shorter regenerating axons in the crush injured sciatic nerve in cKO group. **a**, **b** Western blots and quantification showing the levels of GAP43 in the injured nerve at 3 dpi. **c** GAP43 immunohistochemistry in the longitudinal sections of the injured nerves. Scale bars: 400 µm. **d** The length of the longest GAP43 positive axons from the crush injured site (labeled with yellow line). **e** The number of GAP43 positive axons crossing the lines at 2 mm, 4 mm, 6 mm distal to the injury site. Data are expressed as the mean ± SEM (*n* = 6 per group)

To illustrate the effects of macrophage-specific RhoA depletion on nerve regeneration, a conventional sciatic nerve crush injury model was established in both the cKO and Cre mice. Then Western blotting and immunohistochemistry with the antibody of GAP43, a widely used marker for regenerating axons [35, 40, 41] were performed to evaluate the rate of axonal regeneration at 3 dpi. By which, the blots and quantification showed that the level of GAP43 protein in the distal trunk of the injured sciatic nerve was significantly lower in the cKO group than that of the Cre group (*p* < 0.001, Fig. 3a, b). Moreover, the immunohistochemistry revealed that the GAP43 positive axons extending from the crush site to the distal trunk in 3 dpi in the cKO group were significantly shorter than those in the Cre group (Fig. 3c–e). These findings indicate that macrophage-specific RhoA depletion attenuates axonal regeneration.

**Fig. 4:**
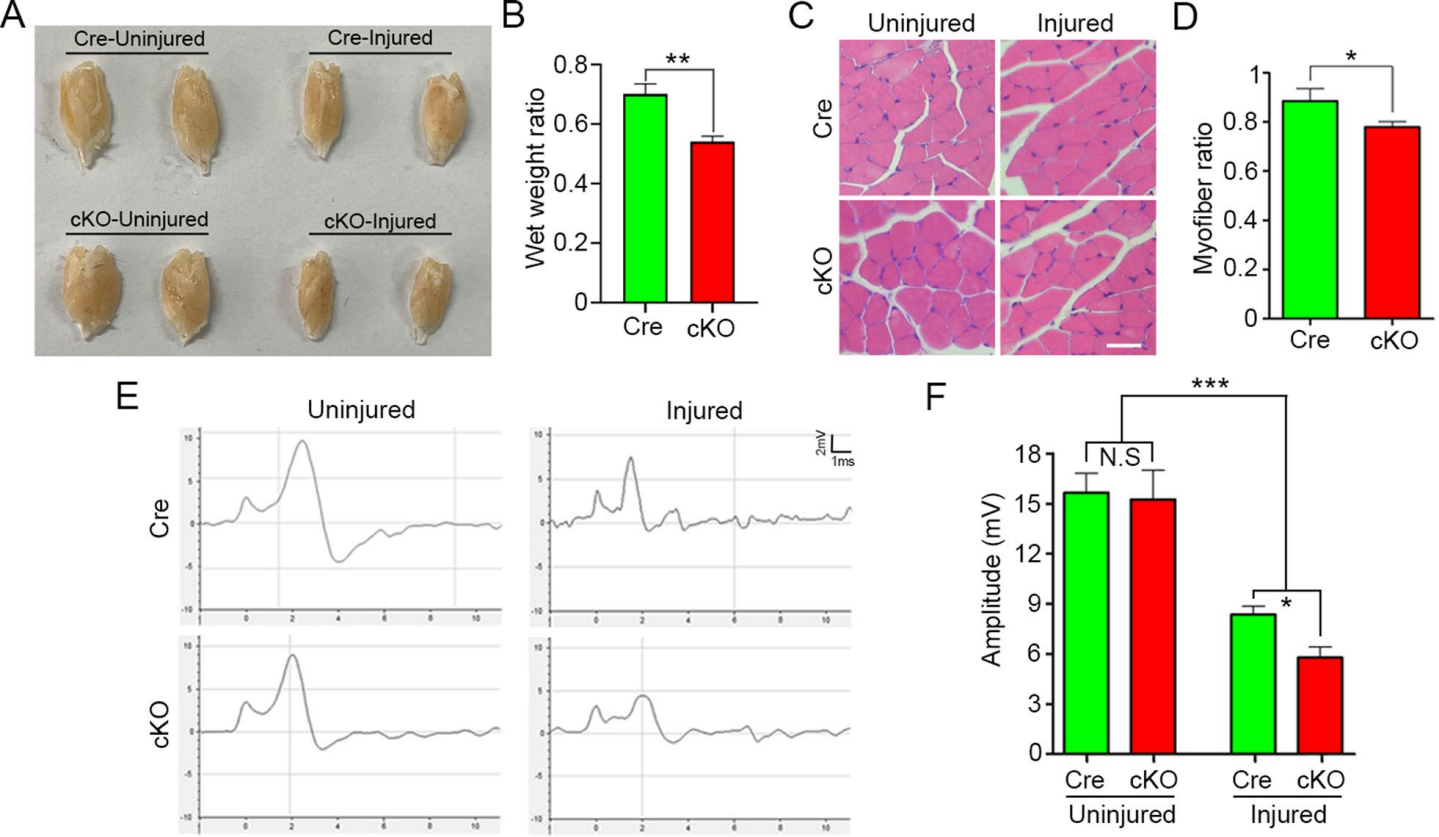
The functional recovery of injured nerve at 28 dpi is worse in the cKO group. **a**, **b** Gloss images and the wet weights of the gastrocnemius muscles. **c**, **d** H&E staining cross sections of gastrocnemius muscles and the ratio of the myofiber area of the injured side compared to the uninjured side. Scale bars: 25 µm. **e**, **f** Electrophysiological records and quantification of the CMAP testing showing no difference between cKO group and Cre group in the uninjured side, but the cKO group has significant lower amplitude than the Cre group in the injured side. Data are expressed as the mean ± SEM (*n* = 6 per group)

According to our previous studies and related literatures [35, 37, 42], mice survived for 28 days after crush and then the wet weight and myofiber area of gastrocnemius muscle as well as the amplitude and CMAP were detected to assess the functional outcomes of nerve regeneration. First of all, the size of the gastrocnemius muscles in the cKO group was obviously smaller than that in the Cre group, and the statistics showed the wet weight of the muscles was lighter in the cKO group than that in the Cre group (*p* = 0.002, Fig. 4a, b). Also, histological analysis of gastrocnemius muscles showed that the level of myoatrophy in the cKO group was worse than that in the Cre group (Fig. 4c, d). Then, the CMAP assaying revealed that the amplitude of cKO group was significantly lower than that of Cre group (Fig. 4e, f). Overall, above results hint that macrophage-specific RhoA depletion delays nerve regeneration after PNI.

### RhoA knockout attenuates the migration and phagocytosis of macrophages

**Fig. 5:**
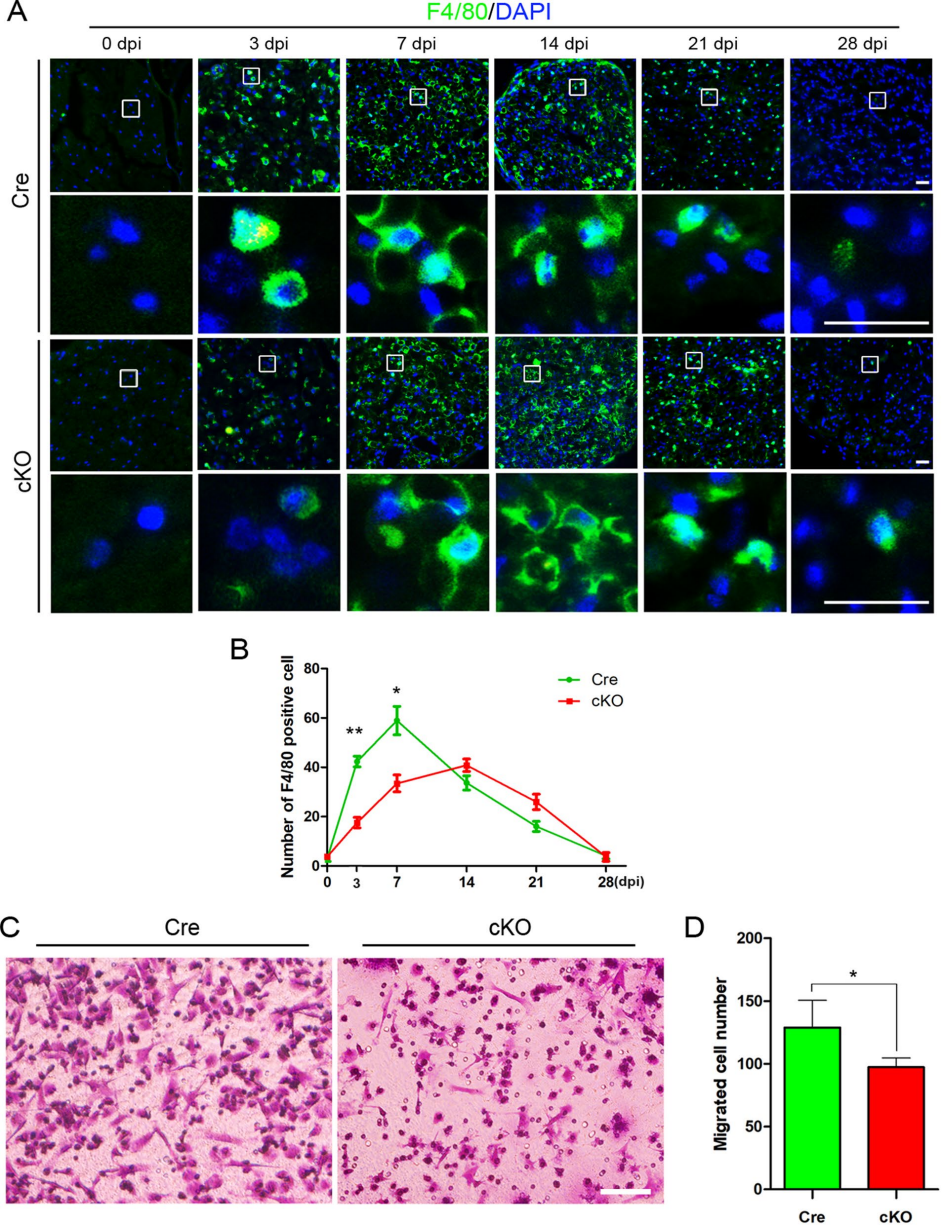
RhoA cKO inhibits the migration of macrophages in vivo and in vitro. **a** F4/80 immunohistochemistry in the cross sections of crush injured sciatic nerve’s distal trunk at 0, 3, 7, 14, 21, 28 dpi. Scale bars: 50 µm. **b** Quantification of the number of macrophages in each time point. **c**, **d** Transwell assay and quantitative analysis showing the number of migrated macrophages in cKO group is significantly less than Cre group in vitro. Scale bars: 100 µm. Data are expressed as the mean ± SEM (*n* = 6 per group)

It is well known that very few resident macrophages in the intact nerve and their phagocytic and proliferation ability were limited [43, 44]. Abundant blood-derived macrophages will be recruited and migrate into the injured nerve to engulf and remove the debris of axons and myelin to play critical roles in the Wallerian degeneration and thus promote nerve regeneration [45, 46]. Therefore, disrupting the migration and phagocytosis of macrophages is most likely the main cause of delayed Wallerian degeneration and nerve regeneration in macrophage-specific RhoA knockout mice. To verify this hypothesis, the macrophages in the injured nerves were detected with F4/80 immunostaining at different times after crush. In the Cre group, the number of macrophages increased dramatically at 3 dpi, reached a peak at 7 dpi. However, the recruitment of macrophages in the cKO group is prolonged with the peak time at 14 dpi, and the number of macrophages at the peak time is significantly smaller than that of the Cre group (Fig. 5a, b). Moreover, present data revealed that the number of macrophages was significantly decreased in the late stages of injury in both Cre group and cKO group, which indicate the macrophage efflux occurred in the late stages. Images in Fig. 5 illustrate that the macrophage efflux is delayed in the cKO group. Since recruitment and efflux of macrophage are highly depending on the capability of cell migration, we also performed the in vitro Transwell assay to detect the difference in migration between two groups. As expected, the number of migrated macrophages was significantly decreased in the cKO group (*p* = 0.15, Fig. 5c, d).

**Fig. 6:**
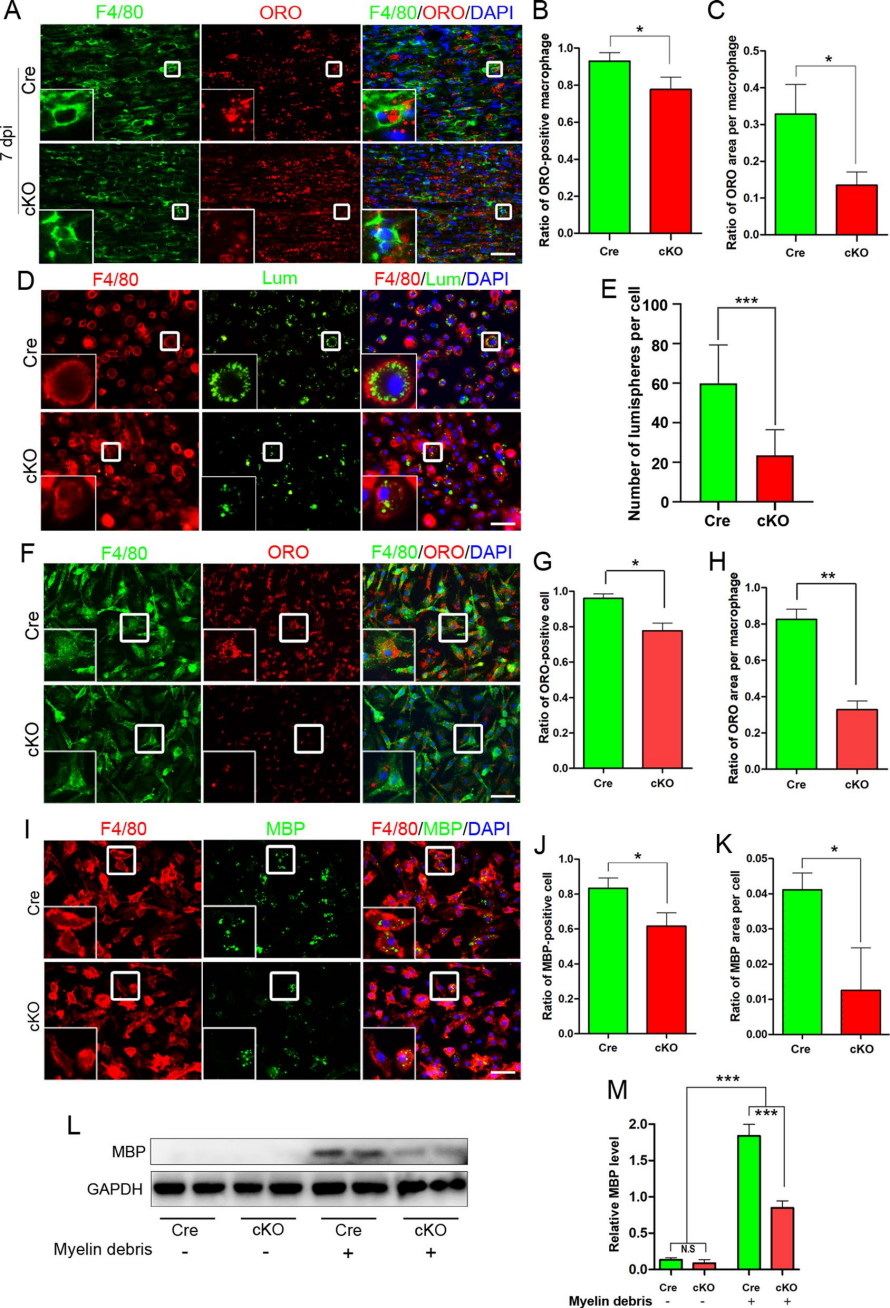
RhoA cKO inhibits the phagocytosis of macrophages in vivo and in vitro. **a**–**c** F4/80 and ORO double staining in the crush injured sciatic nerve at 7 dpi showing that the ratio of ORO^+^ macrophages and the ORO^+^ area in each macrophage are both significantly decreased in the cKO group comparing to the Cre group. Scale bars: 50 µm. **d**, **e** F4/80 immunocytochemistry with the fluorescent lumispheres and quantitative analysis of the number of lumispheres per macrophage in vitro. Scale bars: 50 µm. **f**–**k** F4/80 and ORO **f**–**h**/MBP **i**–**k** double immunocytochemistry staining and quantitative analysis after the macrophages cultured with myelin debris in vitro. Scale bars: 50 µm. **l**–**m** Western blots and quantification showing that MBP expression in cKO group cultured with myelin debris is less than those in Cre group in vitro. Data are expressed as the mean ± SEM (*n* = 6 per group)

Next, we focused on phagocytosis, another important biological function of macrophages [47–49]. At 7 dpi, F4/80 and ORO staining were performed to show macrophages and myelin debris, respectively, in the injured nerve. Statistical data showed that the percentage of macrophages engulfed with ORO^+^ myelin debris in cKO nerves was significantly lower than that in Cre nerves (Fig. 6a). In addition, the engulfed ORO^+^ myelin debris droplet in macrophage was significantly smaller in cKO nerves (Fig. 6b, c).

In order to illustrate the role of RhoA in phagocytic ability of macrophages more clearly, fluorescent lumispheres were added to the culture macrophages to assay the phagocytosis in vitro (Fig. 6d). Quantification showed that the number of lumispheres in each macrophage of the cKO group was significantly less than that of the Cre group (*p* < 0.001, Fig. 6e). Moreover, myelin debris prepared from brain and spinal cord were added into the culture medium of macrophages to mimic the in vivo situation of macrophages devouring myelin debris. Both ORO staining (to show myelin lipid) and myelin basic protein (MBP) immunostaining (to show myelin protein) indicated that the ratio of myelin containing macrophages as well as the phagocytized myelin debris in each macrophage were decreased in the cKO group compared to those in the Cre group (Fig. 6f–k). What’s more, Western blotting also illustrates that less MBP protein in the cKO group (Fig. 6l). Since the macrophages do not express MBP, the level of MBP can be used to reflect the amount of myelin debris phagocytized in the macrophages (Fig. 6m).

### RhoA regulates the migration and phagocytosis of macrophages through ROCK/MLCK pathway

**Fig. 7:**
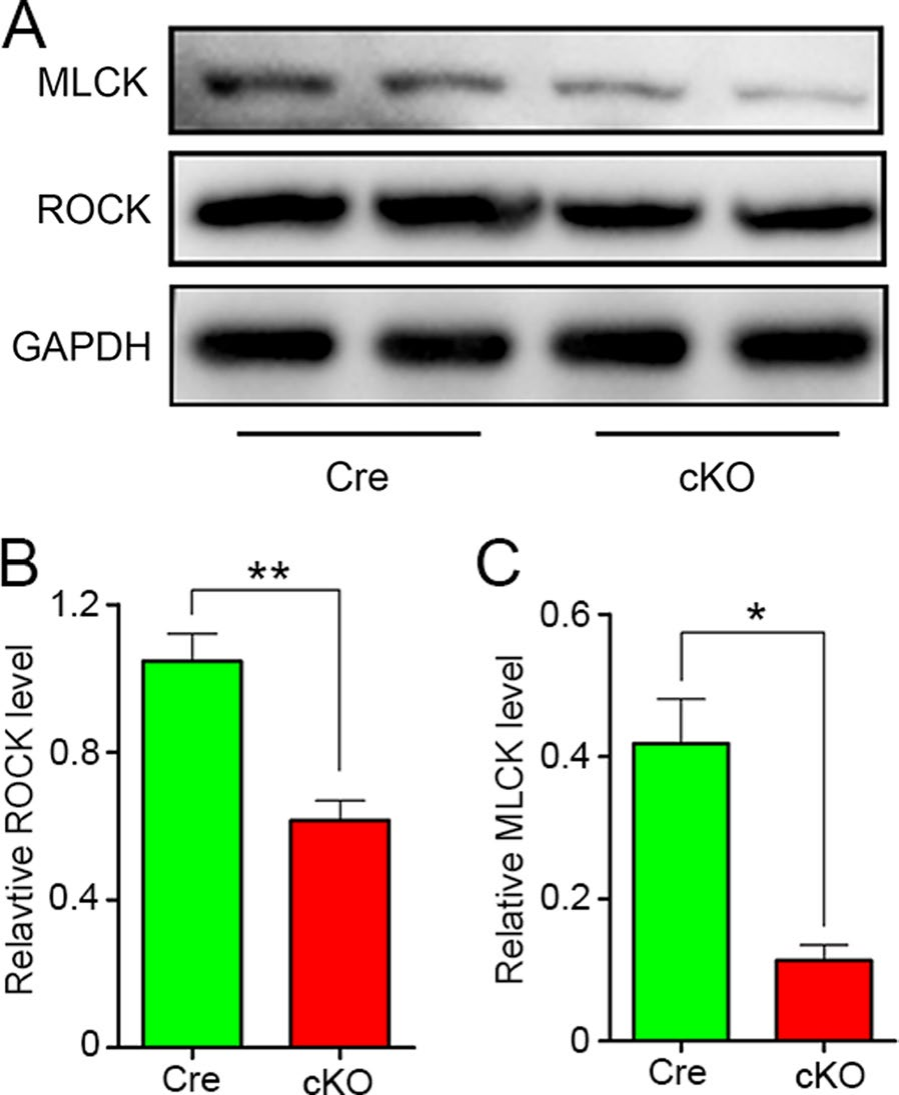
RhoA cKO in macrophages inhibits the expression of ROCK and MLCK. **a** Western blots illustrate that deletion of RhoA in macrophages reduces the protein level of ROCK and MLCK compared to Cre group. **b**, **c** Statistical analysis of the ROCK and MLCK protein level. Data are expressed as the mean ± SEM (*n* = 6 per group)

**Fig. 8:**
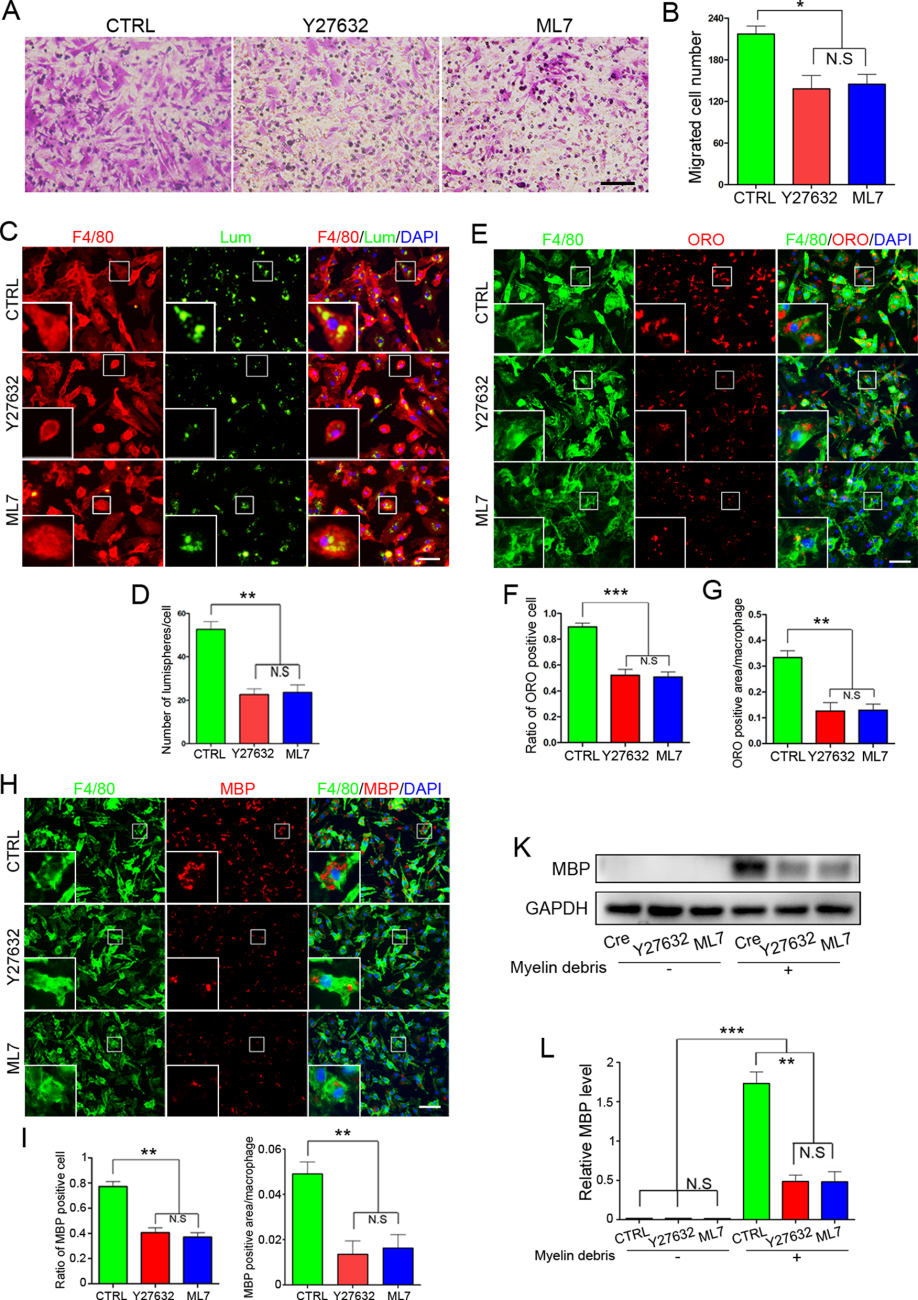
Y27632 and ML7 treatment inhibit macrophage migration and phagocytosis in vitro. **a**, **b** Transwell assay and quantitative analysis showing the number of migrated macrophages from Y27632 group and ML7 group is significantly less than the control (CTRL) group. Scale bars: 100 µm. **c**, **d** F4/80 immunocytochemistry with the fluorescent lumispheres and quantitative analysis. Scale bars: 50 µm. **e**–**j** F4/80 and ORO. **e**–**g **/MBP. **h–j** Double immunocytochemistry staining and quantification of the ratio of ORO^+^ macrophages and the ORO^+^ area in each cell after the macrophages cultured with myelin debris in vitro. Scale bars: 50 µm. **k**–**l** Western blots and quantification showing that MBP expression in Y27632 group and ML7 group cultured with myelin debris are less than those in CTRL group in vitro. Data are expressed as the mean ± SEM (*n* = 6 per group)

RhoA is well known for its importance in many cell types by regulating the cytoskeleton remodeling through ROCK/MLCK pathway [30, 50]. However, whether RhoA deletion alleviates the macrophage’s migration and phagocytosis through ROCK/MLCK pathway is unknown yet. To figure out this issue, we compared the expression levels of ROCK and MLCK in the isolated macrophages by Western blotting. As expected, both ROCK and MLCK were dramatically decreased after RhoA depletion (cKO group) compared to the control cells (Cre group) (Fig. 7a–c). Next, ROCK-specific inhibitor (Y27632) and MLCK-specific inhibitor (ML7) [51, 52] were individually used to assess their effects on the migration and phagocytosis of macrophages. With the Transwell assay, we found that both Y27632 and ML7 impeded the macrophage’s migration (Fig. 8a, b). The fluorescent lumisphere and myelin debris ingestion tests revealed the phagocytosis ability was also declined by Y27632 or ML7 treatments (Fig. 8c–l). Above data indicate that RhoA knockout can down-regulate the expression of ROCK and MLCK, which results in inhibiting the migration and phagocytosis of macrophages, thus delaying the process of Wallerian degeneration, and ultimately leading to the decline of nerve regeneration after PNI.

## Discussion

Previous studies have widely emphasized the therapeutic effects of inhibition of RhoA pathway on promoting peripheral nerve regeneration, and this view is primarily based on the evidences of axonal regeneration after the injured nerves or neurons are treated with down-regulation or inhibition of RhoA pathway [9, 53]. However, recent studies hint RhoA inactivation might be a double-edged sword in regeneration, such as RhoA inhibition in astrocytes has a detrimental effect on axon regeneration [54]. Joshi’s study indicates even motor and sensory neurons respond differently to RhoA inactivation [55].These evidences remind us to play attention to realize that the role of RhoA might be different in various cells. In peripheral nerve, besides neurons, other cells such as Schwann cells and macrophages also play important roles in nerve regeneration [56, 57]. For instance, the Schwann cells in the injured nerve undergo demyelination, dedifferentiation, proliferation, migration, and then form Büngner bands which conduct axonal regeneration. After PNI, an enormous number of macrophages can immigrate from blood or connective tissue into the injured nerve to remove the axonal and myelin debris to create a favorable microenvironment for nerve regeneration [13, 45, 58]. According to the functions of RhoA in neurons, we used to predict that the proliferation, migration, and myelination of Schwann cells might be also promoted by downregulating or depressing RhoA. However, the results are completely different from our expectation [8, 10]. All these draw us to wonder what will happen in the injured nerve when the RhoA pathway is downregulated in macrophages. To figure out this issue, specific conditional knockout RhoA in macrophages should be the most convincing way which can avoid the interference from the changes of RhoA expression in neurons and/or Schwann cells. Therefore, the *Lyz2*^*Cre*^ mice and *RhoA*^*flox/flox*^ mice were used to develop macrophage-specific RhoA cKO mice for this project.

**Fig. 9:**
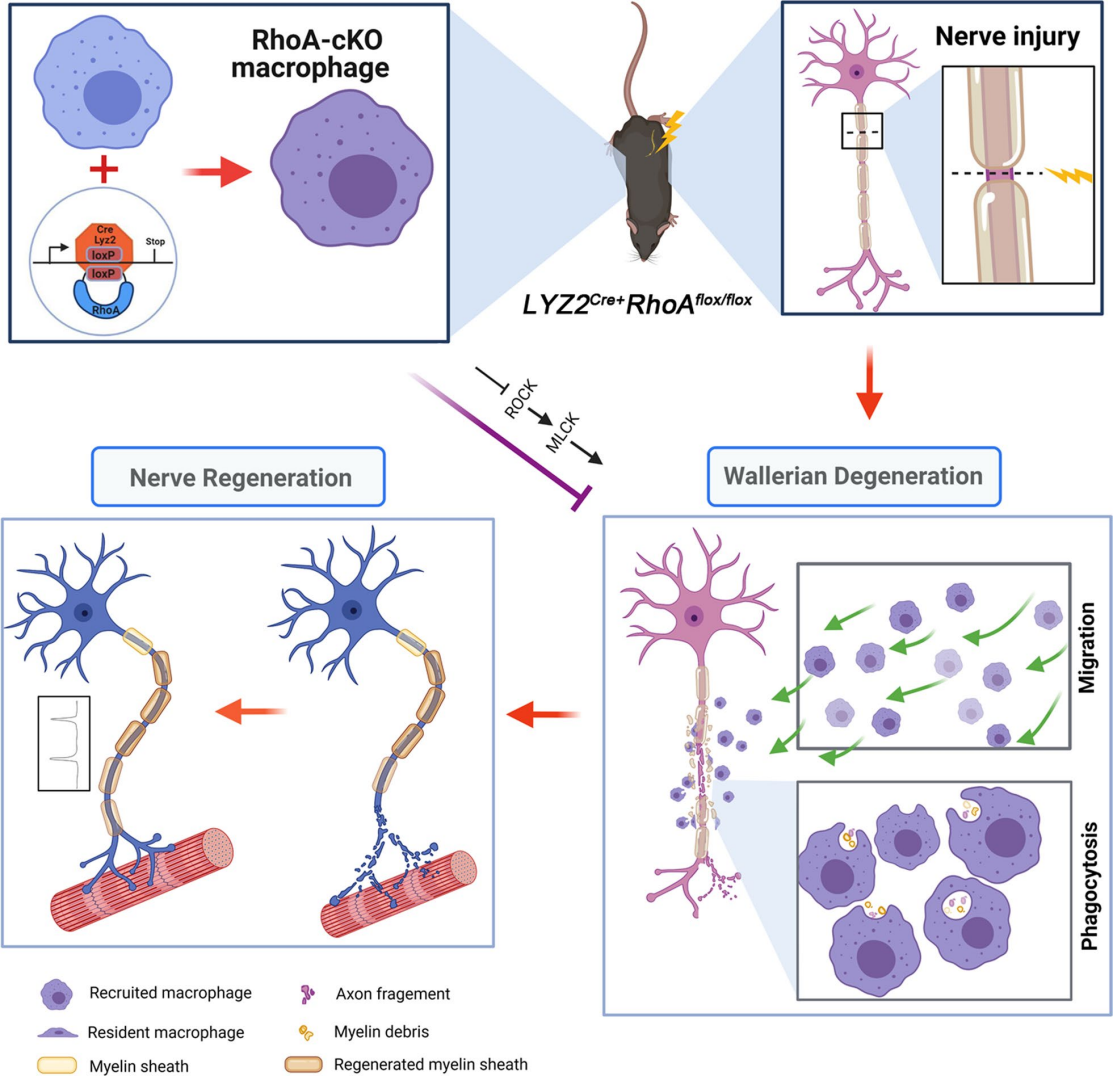
Macrophage-specific RhoA knockout delays Wallerian degeneration and nerve regeneration by impeding macrophage’s migration and phagocytosis

We figured out that the number of exogenous macrophages infiltrating into the injured nerve was relatively small and the recruitment and efflux were delayed in the cKO group. This decreased migration ability of the cKO group was also verified by migration assay in vitro*.* Moreover, we found the phagocytosis ability was decreased in the RhoA cKO macrophages compared with that in the Cre group in vivo, revealed by phagocytosis of both fluorescent microspheres and myelin debris. Actually, the migration and phagocytosis of macrophages necessitate sequential attachment and detachment/retraction of cell tail from the surface. By controlling cytoskeleton reorganization, RhoA pathway modulates the macrophage’s attachment/detachment process and sequentially assembles and disassembles actin and vinculin-rich focal adhesions [59]. Therefore, when the RhoA is knocked out, macrophages migration and phagocytosis are dampened and their consequent functions in the injured nerve might be affected (Fig. 9).

It is well known that the clearance of the axonal and myelin debris mainly depends on the recruited macrophages although the dedifferentiated Schwann cells can also clear parts of debris [32]. The influence on migration and phagocytosis is highly likely to cause changes in the progression of Wallerian degeneration. To assess the process of Wallerian degeneration more accurately, we detected the axon and myelin remained in the distal segment of transected sciatic nerve. In this way, the regenerating axons and myelin extending from the proximal nerve into the distal trunk, which occurs in most PNI models, could be avoided [36, 60]. From the results of Western blots and immunofluorescence, we found that much more axonal and myelin fragments, as well as their proteins, remain in the cKO group which indicated RhoA conditional knockout in macrophages results in the delayed process of Wallerian degeneration.

Since the clearance of axonal and myelin debris is a prerequisite for nerve regeneration [61], the delayed Wallerian degeneration should impede the subsequent nerve regeneration. To verify this hypothesis, we detected the rate of axonal regeneration in the early stage (3 dpi) as well as the functional recovery in the target muscle and nerve conduction capacity using the sciatic nerve crush injury model. At 3 dpi, the protein level of GAP43 and the length of GAP43^+^ axons in the distal injured nerve were detected. GAP43 is a protein specifically expressing in the developing or regenerating axons [62] and is widely utilized to assess axonal regeneration after PNI [35, 63–65]. By this way, we realized that the axonal regeneration is slower in the cKO group. Subsequent assays at 28 dpi, we found that the wet weight of gastrocnemius muscle and the size of its myofibers, as well as the amplitude of CMAP of electrophysiological testing, are all significantly decreased in the cKO group. These results felt very reasonable as they are consistent with the macrophages’ declined capabilities of migration and phagocytosis and the prolonged process of Wallerian degeneration.

It is generally considered that the most important event during the Wallerian degeneration is to engulf and remove the debris of axons and myelin, and this task relies heavily on macrophages. In peripheral nerve, there are some resident macrophages which will undergo activation, proliferation, and phagocytosis within 2 days after nerve injury [43]. However, the contribution of these resident macrophages for the debris clearing is limited. Perry’s study had showed the denervated Schwann cells, but not the resident macrophages, are the major phagocytic cells for the first 5 days after nerve injury [66]. In this early stage, the denervated Schwann cells release many cytokines that act as chemoattractants [67, 68], then, massive exogenous macrophages are recruited from circulating blood in the later stage (from 4 days after nerve injury), and work as main phagocytic cells to the clearance of debris in later stage of the Wallerian degeneration [46]. Actually, the macrophage recruitment (including chemoattractant and migration), and the phagocytosis, all rely on the movement of macrophages forward [69], which have an indiscerptible association with cytoskeletal remodeling. Numerous studies have shown that RhoA is a key regulator for cytoskeletal remodeling in various cells via its downstream ROCK/MLCK pathway [70, 71]. However, whether this mechanism also existed in macrophages migration and phagocytosis is unknown yet. Therefore, we conducted a series of experiments illustrating that RhoA deletion can dramatically decline the ROCK and MLCK expression in macrophages. Furthermore, both ROCK or MLCK inhibitors were found can alleviate the macrophage’s capacities of migration and phagocytosis.

## Conclusion

As illustrated in Fig. 9, collective data of present study indicated that RhoA cKO in macrophage may affect its migration and phagocytosis via ROCK/MLCK pathway, delay the process of Wallerian degeneration and result in the alleviated nerve regeneration. Even previous studies have demonstrated that RhoA inhibition can promote peripheral nerve regeneration; it should be noticed that various cells in the injured nerve have different responses to the same treatment. Targeted drug delivery to different cells in the future molecular therapy for PNI is strongly suggested when any drug is used to regulate RhoA pathway.

## Data Availability

All data generated or analyzed during this study are included in this published article.

## Abbreviations

PNI: Peripheral nerve injury
RT: Room temperature
ORO: Oil Red O
PVDF: Polyvinylidene difluoride membrane
CMAP: Compound muscle action potential
H&E: Hematoxylin–eosin
SEM: Standard error of the mean
MBP: Myelin basic protein
